# Adaptive goal setting and financial incentives: a 2 × 2 factorial randomized controlled trial to increase adults’ physical activity

**DOI:** 10.1186/s12889-017-4197-8

**Published:** 2017-03-29

**Authors:** Marc A. Adams, Jane C. Hurley, Michael Todd, Nishat Bhuiyan, Catherine L. Jarrett, Wesley J. Tucker, Kevin E. Hollingshead, Siddhartha S. Angadi

**Affiliations:** 1 College of Health Solutions, Arizona State University, 425 North 5th Street (MC9020), Phoenix, AZ 85004 USA; 2 College of Nursing and Health Innovation, Arizona State University, 500 N. 3rd Street, Phoenix, AZ 85004 USA; 3 Global Institute of Sustainability (GIOS), Arizona State University, Tempe, AZ 85287 USA

**Keywords:** Adaptive interventions, Reward, Goals, mHealth, Pedometer, Fitbit, Text messaging

## Abstract

**Background:**

Emerging interventions that rely on and harness variability in behavior to adapt to individual performance over time may outperform interventions that prescribe static goals (e.g., 10,000 steps/day). The purpose of this factorial trial was to compare adaptive vs. static goal setting and immediate vs. delayed, non-contingent financial rewards for increasing free-living physical activity (PA).

**Methods:**

A 4-month 2 × 2 factorial randomized controlled trial tested main effects for goal setting (adaptive vs. static goals) and rewards (immediate vs. delayed) and interactions between factors to increase steps/day as measured by a Fitbit Zip. Moderate-to-vigorous PA (MVPA) minutes/day was examined as a secondary outcome.

**Results:**

Participants (*N* = 96) were mainly female (77%), aged 41 ± 9.5 years, and all were insufficiently active and overweight/obese (mean BMI = 34.1 ± 6.2). Participants across all groups increased by 2389 steps/day on average from baseline to intervention phase (*p* < .001). Participants receiving static goals showed a stronger increase in steps per day from baseline phase to intervention phase (2630 steps/day) than those receiving adaptive goals (2149 steps/day; difference = 482 steps/day, *p* = .095). Participants receiving immediate rewards showed stronger improvement (2762 step/day increase) from baseline to intervention phase than those receiving delayed rewards (2016 steps/day increase; difference = 746 steps/day, *p* = .009). However, the adaptive goals group showed a slower decrease in steps/day from the beginning of the intervention phase to the end of the intervention phase (i.e. less than half the rate) compared to the static goals group (−7.7 steps vs. -18.3 steps each day; difference = 10.7 steps/day, *p* < .001) resulting in better improvements for the adaptive goals group by study end. Rate of change over the intervention phase did not differ between reward groups. Significant goal phase x goal setting x reward interactions were observed.

**Conclusions:**

Adaptive goals outperformed static goals (i.e., 10,000 steps) over a 4-month period. Small immediate rewards outperformed larger, delayed rewards. Adaptive goals with either immediate or delayed rewards should be preferred for promoting PA.

**Trial Registration:**

ClinicalTrials.gov ID: NCT02053259 registered prospectively on January 31, 2014.

## Background

American men and women are insufficiently physically active [1, 2], with little change in population levels of physical activity (PA) over the past two decades [3, 4]. This trend has led to a large body of studies focused on increasing PA using diverse strategies, theories, and models for behavior change. A meta-analysis (*N* = 99,001, 358 papers) of individual-level interventions designed to increase PA among healthy adults using a variety of motivational strategies found an overall mean difference of just below 500 steps per day (or 2.1 min/day), favoring intervention over control groups [5]. Because the majority of US adults accumulate less than 7500 steps per day [6, 7] and less than 6 min of moderate-to-vigorous physical activity per day [1], the aforementioned review indicates a dire need for more potent intervention strategies and treatments to increase and sustain adults’ physical activity to levels of at least 10,000 steps or 30 min per day [5].

Physical activity behavior is highly variable within individuals over time. In an observational study, Rowlands et al. reported high levels of intra-individual variability in day-to-day steps over 1 year, and argued that interventions will need to start accounting for day-to-day fluctuations to promote and sustain physical activity [8]. Currently, many research interventions and commercial programs aimed at increasing PA among the public often prescribe fixed criterion targets (e.g., 10,000 steps or 30 min per day) [9], or offer goals that increase linearly by some fixed amount over the course of an intervention (e.g., 5–10% or 250 steps/week) [10–12]. Such relatively static intervention components can be insensitive to daily intra-individual variability and fail to respond to individuals: some people change quickly, whereas others change slowly during an intervention. Behavior change interventions that *adapt* frequently (e.g., daily) and uniquely to individual performances over time may hold promise for enhancing PA adoption and maintenance [8, 13–15]. Adams et al. tested a new approach to goal setting that adjusted step goals up or down using a percentile-rank approach based on an individual’s ongoing performance. The authors found a difference of 1130 steps per day using multi-component intervention that included adaptive goals and immediate financial rewards compared to individuals receiving static goals (i.e., 10,000 steps per day) combined with delayed financial rewards for ongoing study participation [16]. The study also demonstrated that adaptive goals with immediate financial rewards reduced intra-individual variability in steps over 6 months. Given existing evidence and theoretical support that behavior change is not a rational, linear, or even threshold process, offering criterion targets as goals may not be the most effective strategy [2, 16, 17].

Basic principles of positive reinforcement [18, 19] and behavioral economics [20–25] are integrated into many theoretical approaches (e.g., Transtheoretical Model [26, 27], Social Cognitive Theory [28, 29], and Ecological Models [30, 31]) and have been proposed as unifying behavior change principles behind preventive medicine [32]. Positive reinforcement should be preferred because gain-based approaches produce fewer psychological side effects (e.g., aggression, frustration) than approaches based on aversive control such as penalties (e.g., loss of reward) [33] and may be more effective than non-contingent or interval-based (delayed) reinforcement for long-term behavior change [20, 31, 32]. However, most research on incentives for physical activity has tested deposit contracts (a loss-based approach) or focused on reward magnitude (amount) as dimensions of incentive architecture, mainly for exercise session attendance [34], with recent exceptions [16, 35, 36] targeting steps/day. Operant and behavioral economic models argue that because behavior change is not rational, smaller more immediate positive rewards that engage individuals more frequently can be used to help shape improvements in activity over time. Combining adaptive goals and immediate rewards for goal attainment requires frequent monitoring of behavior (and variability) to capture and reinforce improvements to encourage better performance. Principles of shaping can now be combined with ubiquitous mobile technologies, such as text messages and internet-connected activity monitors, to capture and reward improvements in near-real time for physical activity with higher frequency and precision than has been accomplished in past studies [37–39].

The current efficacy study builds on previous work on adaptive goals and immediate positive reinforcement to shape increased levels of physical activity [16, 40]. In our previous research, a multicomponent intervention that combined adaptive goals with immediate rewards outperformed static goals with delayed rewards, but questions remained about whether observed effects were attributable to goal setting or reward components or both. In the current 2 × 2 factorial randomized controlled trial (RCT), we tested these components for their independent and joint contributions on steps/day using Fitbits. A factorial trial is an efficient design for untangling multicomponent interventions and theoretical mechanisms by design [41, 42]. We hypothesized that participants receiving adaptive goals would increase their steps/day compared to static goals; participants receiving immediate rewards would increase steps/day more than rewards for study participation; and participants receiving adaptive goals with immediate rewards would outperform the other combination groups by the end of the study. Secondary aims were to test for differences across arms in participants’ moderate-to-vigorous PA (MVPA).

## Methods

**Fig. 1:**
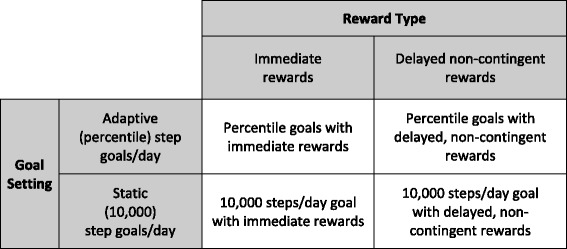
Illustration of the 2 × 2 factorial design for WalkIT with goal setting and reward type factors

The study rationale, design and measures for the Walking Intervention Through Texting (WalkIT) trial have been described previously by Hurley et al. [39]. Briefly, a 4-month 2 × 2 factorial RCT was used to test for main effects for goal setting (adaptive vs. static goals) and financial rewards (immediate rewards for PA goal attainment vs. delayed rewards for study participation) and interactions between factors to increase pedometer-measured steps/day (see Fig. 1). Financial rewards for the delayed group were provided on an escalating monthly (interval) schedule for ongoing study participation. These rewards were considered delayed and non-contingent for PA relative to the immediate rewards group because they were contingent on ongoing study participation (henceforth labeled “delayed reward” group). Immediate rewards were contingent on meeting daily PA goals. All participants were provided a blinded Fitbit Zip (Fitbit Inc., San Francisco, CA, USA) at the baseline office visit and instructed to maintain their usual PA routine over the next 10 days. This 10-day baseline phase acted as a lead-in to ensure minimal adherence to study protocol, objectively verify insufficient levels of activity (i.e., did not achieve ≥10,000 steps/day on ≥5 days/week), and ensure compatibility of the participant’s computer with Fitbit software. Participants were considered eligible for randomization after wearing the Fitbit for at least 9 valid days (valid day required ≥500 steps) and syncing it successfully. This approach ensured that participants had compatible computers at home, reactivity to the device subsided [43], and sufficient data were available from the participant to start the adaptive goal setting algorithm (see experimental component 1 below). Participants who completed the baseline phase were immediately randomized into one of the four 110-day interventions. The intervention components were delivered primarily by text message (i.e., SMS). The Institutional Review Board at Arizona State University approved the registered trial ClinicalTrials.gov ID: (NCT02053259) and all study procedures were carried out in accordance to the declaration of Helsinki.

### Recruitment and participants

Individuals were recruited from using flyers and email announcements posted in local business and community settings, on social media, and sent to listservs affiliated with government, business and special interest groups. Recruitment materials invited individuals to join a 4-month intervention to increase physical activity and guided them to an online pre-screening survey that evaluated inclusion/exclusion criteria for each respondent.

### Inclusion and exclusion criteria

Generally healthy, overweight/obese, inactive and insufficiently active adult men and women between 18 and 60 years old were invited to participate. Overweight and obese adults were the target population because they are less likely than their normal weight peers to meet physical activity guidelines when measured by either self-report or accelerometry, [44] and can benefit from improving energy-balance behaviors. Body mass index (BMI) was initially assessed by self-reported height and weight (later measured at the office visit), and individuals within 25 and 55 kg/m^2^ were contacted. Participant activity/inactivity status was assessed online during the pre-screening survey which included the International Physical Activity Questionnaire (IPAQ) short form, and again after enrollment but prior to randomization during the 10-day baseline period monitored with a blinded Fitbit Zip. To be eligible for randomization, inactive and insufficiently active participants were defined as those that did not achieve ≥10,000 steps/day on ≥5 days/week as measured by a Fitbit Zip. Individuals were excluded if they (a) lived outside of the study area, (b) reported a medical condition on the Physical Activity Readiness Questionnaire plus (PAR-Q+) that contraindicated unsupervised exercise or submaximal exercise testing, (c) planned to become pregnant in the next 4 months, (d) planned to leave the study region for more than 10 days in the next 4 months, (e) were actively participating in another physical activity, diet, or weight loss program, (g) did not have daily access to Windows or Mac computer, daily access to email and the internet, or a mobile phone with text messaging capabilities, or (h) were unwilling to send and receive several text messages daily.

During the initial visit participants completed a written informed consent form, the PAR-Q, and measures of demographic, personal, and psychosocial characteristics. Project staff directly measured height and weight (seca 284, Germany) and trained participants on the Fitbit Zip and the texting system. At a 4-month follow-up visit, each participant returned the Fitbit Zip, completed post-intervention measures, and was debriefed regarding the study purpose. Data collection staff were blinded to treatment allocation at pre- and post-intervention assessments. Participants, however, were not blinded to treatment as the post-randomization emails described what to expect in each group during the intervention phase.

#### Intervention components

All participants were told that they would receive one of four physical activity interventions that included similar components including Fitbits, text messages, goal setting, and incentives. After randomization, participants in all groups received an email that encouraged them to strive for an ultimate target of 10,000 steps on ≥5 days/week, and included two brochures on physical activity: “Be Active Your Way: A Guide for Adults” published by the U.S. Health and Human Services [45] and “100 Ways to Add 2000 Steps” published by the America on the Move Foundation [46].

Aside from brief educational materials, components used in the four interventions were delivered via a semi-automated text message system developed by our study team. Participants received one daily prompt-to-action message that included either tips, questions, motivational or inspirational sayings. For example, “It doesn’t matter how old you are – it’s never too early or too late to become physically active so start today; only then will you start to see results!” and “Step tip: You don't have to exercise by yourself. Take a pet or encourage friends and family members to be physically active with you.” A pool of messages developed for the study were randomly shuffled for each participant and sent at a random time between 8 AM and 6 PM each day.

During the intervention phase, participants were asked to monitor their accumulated steps using their unblended Fitbit and text their steps, called a step report, by midnight (e.g., 5580 steps) or early the next morning (e.g., 5580 yesterday) to the study texting system designed for limited natural language recognition, which monitored, acknowledged, and responded to step reports within 30 s to 3 min by providing differential feedback based on condition and goal attainment. Any participant-generated text messages that the natural language recognition system did not recognize as a step report were forwarded to researchers on call. Goal setting and reward delivery functions were controlled by a programmed automated system. Participants typically sent and received a total of 2–3 text messages daily.

#### Experimental component 1: adaptive vs. static goal setting

In the adaptive goal group, daily goals were based on each individual’s unique performance using a moving-window percentile-rank algorithm. The 10-day baseline phase was used to calculate the first adaptive goal after ignoring the first day. The percentile-rank approach requires: a) repeated measurements of physical activity, b) ranking of steps/day from lowest to highest, and c) calculation of a new goal based on a nth percentile criterion. For example, for a single participant, daily step count over the last 9 days (ranked from lowest to highest) was 1250, 1332, 3136, 5431, 5552, 5890, 6402, 7301, 10,103. In this case, the 60th percentile represents a goal of 5890 steps. This value was rounded up to the nearest multiple of 25 steps, or 5900 steps, which became the next day’s goal. The moving window incorporated each new day’s steps: newest step count replaces the oldest step count observation. The 60th percentile approach offers a standardized, scalable, and generalizable approach to personalized goal setting across participants, but with step/day values unique to individual performances. Previous research by Adams et al. [16, 40] suggested a 60th percentile value for the current study. Participants were informed that adaptive goals were valid for a single day only and subsequent goals could stay the same, decrease, or increase. This approach encouraged participants to text us their daily step reports unprompted. The most recent 9 consecutive days of non-missing observations were used when missing step data were observed during the intervention phase. In the static goal condition, the standard recommendation of 10,000 steps/day over 5 out of 7 days (as described in the initial email sent to all groups) was prescribed. Regardless of the group, our system was programmed to text the next step goal each time a participant reported their steps via text in the evening or early morning, or automatically whenever a participant requested a “goal reminder” by texting “goal”.

#### Experimental component 2: immediate vs. delayed, non-contingent rewards

Each time a participant texted a step report, the system provided feedback based on reward group assignment. Participants in the immediate reward group received differential feedback contingent on meeting a daily step goal. For example, when a goal was met, participants received a text with a praise message, and their point balance (e.g., “Well done, John! You have 3 points! Your goal for 3/16/2014 is *[…]* steps.”). When a goal was not met, a confirmation of the step report was provided along with the next goal (e.g., “Steps Received. Goal for 3/16/2014 is *[…]* steps”), acknowledging the participant’s text without discouraging feedback. Participants in the immediate reward group were told at randomization that they could earn one point each day they met a step goal; one point equaled $1.00. Each time 5 points were accumulated, points were automatically exchanged for a $5 gift card, which our system sent by email. The 60th percentile of each participant’s measured repertoire ensured that about 40% of goals on average were met over the intervention duration. We used this financial value (i.e., 40% of 110 days =44 x $1.00 = $44.00) to approximate total incentive values for the delayed reward group.

Participants in the delayed reward group did not receive praise messages or points for achieving goals, but were told at randomization they would receive a progressively increasing magnitude of monthly incentives (month 1 = $5; month 2 and 3 = $10 each; month 4 = $20; total $45) for wearing their Fitbit Zip, syncing regularly, and participating in the study, which is similar to how many published trials provide incentives for participation [34]. The study was structured such that participants in both groups received $10 for completing the baseline phase and $15 for completing the 4-month visit, in addition to approximately $44–45 during the intervention phase, for a total of $69–$70 over the entire study. Attempting to match incentive amounts across groups at study onset controlled for the potential confounding of total amount.

Participants selected a preferred incentive type from a list of available retail options (Amazon, iTunes, Target, Walmart, Barnes and Noble, CVS) or a charity (i.e. the United Way) at the baseline visit and participants could request a different option going forward from the incentive list at any time via text or email. The option to change incentive type was offered to participants to reduce the likelihood of satiation (i.e., banking incentives and not needing additional ones) or habituation (i.e., losing motivation due to lack of novel reinforcing stimuli) [47]. Gift cards were emailed at the appropriate time depending on group assignment.

#### Primary and secondary outcome measures

The primary outcome measure was steps per day as measured by the Fitbit Zip. A secondary outcome was moderate-to-vigorous PA (MVPA) minutes per day defined as the sum of daily minutes with a step count (or cadence) of ≥100 steps per minute [48], computed using minute-level epoch data recorded by the Fitbit Zip. Participants were issued a Fitbit Zip, a small tri-axial, hip worn accelerometer, for the 4-month duration of the study. They were instructed to wear it during all waking hours (i.e., ≥10 h) every day during the baseline and intervention phases, removing it only for sleep or before submerging it in water (e.g., prior to showering or swimming). The Fitbit Zip was worn clipped onto clothing near the hip and has an unobtrusive form factor to minimize non-wear. The Fitbit Zip has excellent reliability (ICC = 0.90) [49], and validity for measuring steps in the lab and during free-living conditions (ICCs =0.99–1.0, respectively) compared to direct observation and activPAL (mean absolute percentage error = 0.3% - 1.2%), respectively [49] and compares well to research-grade pedometers. [50]. The Fitbit Zip is also valid for measuring cadence (steps/min) faster than 0.7 m/s (1.57 miles per hour) [51].

Participants were instructed to sync their Fitbit Zip daily using a sync dongle that connected via USB port to a personal computer. Step data were transmitted automatically from the participant’s PC to the Fitbit data collection host, and our system retrieved daily summary and minute-level step data via Fitbit’s commercial API. For goal setting components, participant step reports were used for the most proximal day of data (i.e., current day’s steps) and data obtained from Fitbit replaced the other 8 of 9 observations in the moving-window algorithm. Steps reports were used for current day’s steps instead of data from the Fitbit to ensure that participants who could not sync temporarily (due to traveling or connectivity issues) could still interact with the system (few discrepancies were observed between step reports and data from Fitbits). When a participant failed to sync after 48 h, but texted in a step report, our system would respond to a step report with feedback appropriate to goal attainment status, but our feedback would indicate that the next goal was unavailable until a successful Fitbit sync. For example, “Woohoo! You have 4 points! A goal cannot be provided because your Fitbit has not synced recently. Please sync your Fitbit! Then text GOAL.” This process ensured timely data collection and allowed us to verify texted step reports. The research team created and controlled all Fitbit accounts that were paired with the Zip devices, so participants were not able to access or view activity history, nutrition trackers, “badges” earned, social media interfaces, or any other online tools. This procedure prevented any potential confounding influences from the Fitbit dashboard and precluded confounding influences if Fitbit changed any aspect of their dashboard or system during the study period.

#### Demographic and other variables

Participants reported by survey their age, sex, race and ethnicity, smoking status, employment status (currently employed or not), marital status (married or living with partner vs. other), parental status (one or more children vs none), household income (four levels from <$25,000/year to $75,000–$99,000/year), student status (enrolled in school vs not), and highest educational attainment (three levels from less than high school diploma to 4-year college degree or higher). BMI (kg/m^2^) was determined from researcher-measured height and weight. Meteorological data (minimum and maximum temperature, rain, fog, wind speed) for all possible study days were obtained from National Oceanic and Atmospheric Administration [52].

#### Analytic approach

##### 2.2.6.1.Sample size

As described in detail elsewhere [39], simulations based on effect size estimates derived from previous work revealed that under conservative sets of assumptions (i.e., sets comprising combinations of small effect magnitudes), a total sample of *N* = 80 participants (*n* = 20 per group) would be required to have power of 0.80 or greater to detect hypothesized interaction effects. We oversampled by 20% to account for attrition.

##### 2.2.6.2.Preliminary model specification

Steps per day and MVPA minutes per day were modeled as continuous outcomes using a linear mixed models (LMMs) with repeated observations (*n* = 9825 out of 11,520 planned observations) of each dependent variable (Level 1) treated as nested within (*N* = 96) individual participants (Level 2). The base models included fixed effects for 6 dummy (0, 1) vectors coding for day-of-week and 11 dummy vectors coding for calendar month, a random person-level intercept, and parameters specifying a first-order autoregressive or AR(1) autocorrelation structure among the day-level residuals. Based on results of preliminary model fitting steps, fixed effects for additional background covariates were included at Level 1 (rain and fog) and Level 2 (mean-centered age and BMI, sex, race/ethnicity, marital status, parental status, employment status, and smoking status) in the base model of each outcome.

##### 2.2.6.3.Testing effects of individual intervention components

We added to our models a dummy coded phase (baseline vs. intervention) vector, a post-randomization study day (Intervention Day: all baseline days =0, first post randomization day =1, last study day =110), effect coded (−1, +1) vectors for goal type (static vs. adaptive) and reward schedule (delayed vs. immediate); goal x phase and reward x phase interaction terms (to test for between-group differences in change from baseline to intervention phase); and goal x intervention day and reward x intervention day interaction terms (to test for between-group differences in the rate of change from day 1 to day 110 of the intervention phase). The form of each interaction was determined using methods and tools described and developed by Preacher et al. [53], which yielded values for model-estimated means. 

##### 2.2.6.4.Exploring interactions between intervention components

We explored potential goal x reward interaction effects on change from baseline and to the intervention phase. To do this, a model with goal x reward and goal x reward x phase interaction terms was estimated for each outcome. The significance of the three-way interaction term was determined, and, as with each two-way interaction, its form was explored using methods and tools described and developed by Preacher et al. [53]. As there was insufficient power to examine goal x reward x intervention day interactions (i.e., group x reward interaction effect on the rate of change from day 1 to day 110 of the intervention phase), this interaction was not modeled. An intent to treat approach without imputation was used and included available days of PA data from all participants. To depict typical group trajectories, predicted values for each person-day (measurement occasion) with group-specific, loess-smoothed regression lines were plotted (see Figs. 3, 4, 5 and 6).

## Results

**Fig. 2:**
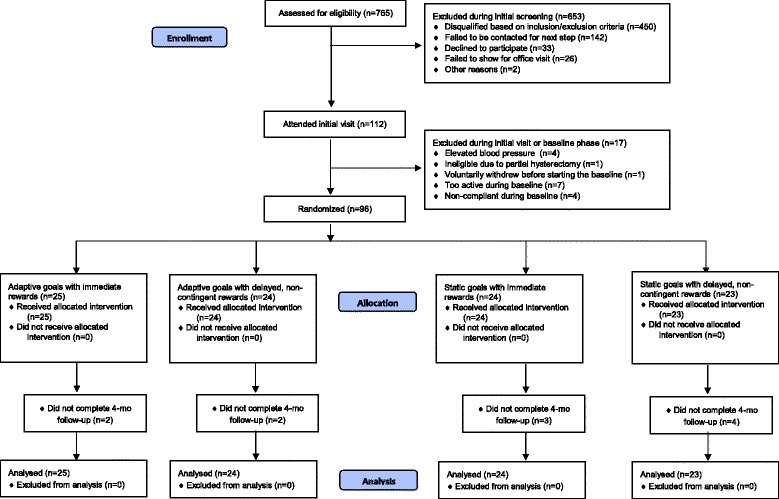
CONSORT Diagram for WalkIT

Figure 2 shows enrollment and participation across groups. Of the 765 individuals screened for eligibility, 112 (14.6%) appeared eligible and attended the baseline visit. The remaining individuals were excluded because they did not meet inclusion/exclusion criteria or could not be contacted. Of the 112 individuals who attended the visit and/or started the 10-day baseline phase, 16 (14.3%) were not randomized for various reasons (see Fig. 2). Those individuals were less likely to identify as White and more likely to identify as African American. Of those who attended an initial office visit, 96 were randomized.

Table 1 presents anthropometric and personal characteristics of the randomized sample. Approximately 77% of participants were female, with a mean age of 41 years (*SD* = 9.5) and a BMI of 34.1 (*SD* = 6.2). Participants were generally well balanced across groups, with a larger proportion identifying as African American and a smaller proportion identifying as White in the static goals with immediate rewards group. Median number of daily step and MVPA observations was 112 and ranged from 108 to 113 across groups.

**Table 1:** Demographics and personal characteristics by group status

	Total (*N* = 96)	Adaptive with Immediate Rewards (*n* = 25)	Adaptive with Delayed Rewards (*n* = 24)	Static with Immediate Rewards (*n* = 24)	Static with Delayed Rewards (*n* = 23)
Age, mean (SD)	41.0 (9.46)	41.0 (10.16)	44.5 (10.70)	38.4 (8.22)	40.3 (7.91)
BMI, mean (SD)	34.1 (6.18)	33.6 (6.31)	33.1 (5.98)	35.1 (5.34)	34.6 (7.20)
Female, %	77.1	88.0	70.8	79.2	69.6
Race and Ethnicity*					
White, %	81.3	84.0	83.3	62.5	95.7
Black, %	9.4	0.0	8.3	29.2	0.0
American Indian, %	4.2	12.0	0.0	0.0	4.3
Asian, %	4.2	0.0	8.3	4.2	4.3
Refuse to answer, %	3.1	4.0	0.0	8.3	0.0
Hispanic, %	19.8	24.0	16.7	25.0	13.0
Mixed Race/Ethnicity, %	20.0	25.0	16.7	20.8	17.4
Smokers, %	9.4	8.0	4.2	4.2	21.7
In School, %	11.5	12.0	8.3	16.7	8.7
Married^a^, %	51.0	64.0	54.2	33.3	52.2
Employed, %	96.9	100.0	100.0	95.8	91.3
Has Children, %	66.3	72.0	60.9	62.5	69.6
# of children, median	2	2	2	2	2
Household Income, median $	$50,000–74,999	$50,000–74,999	$50,000–74,999	$50,000–74,999	$50,000–74,999
Education, median	College graduate	College graduate	College graduate	College graduate	College graduate
# of days with PA data, median	112	113	113	112	108

*SD* standard deviation

*Race/ethnicity cumulative is >100%. Participants were allowed to “select all that apply”

^a^Married includes married and living with partner/significant other

### Effects of individual components on steps per day

Participants on average increased by 2389 steps/day from baseline to intervention phase (b = 2389.38, 95% CI [2102.11, 2676.65], *p* < .001), and during the intervention phase, in addition to an increase in the average level, there was an average decline of 13.0 steps each day (b = −13.01, 95% CI [−19.77, −6.24], *p* < .001) across all groups during the intervention phase, i.e., from 1 day post-randomization to 110 days post-randomization.

**Fig. 3:**
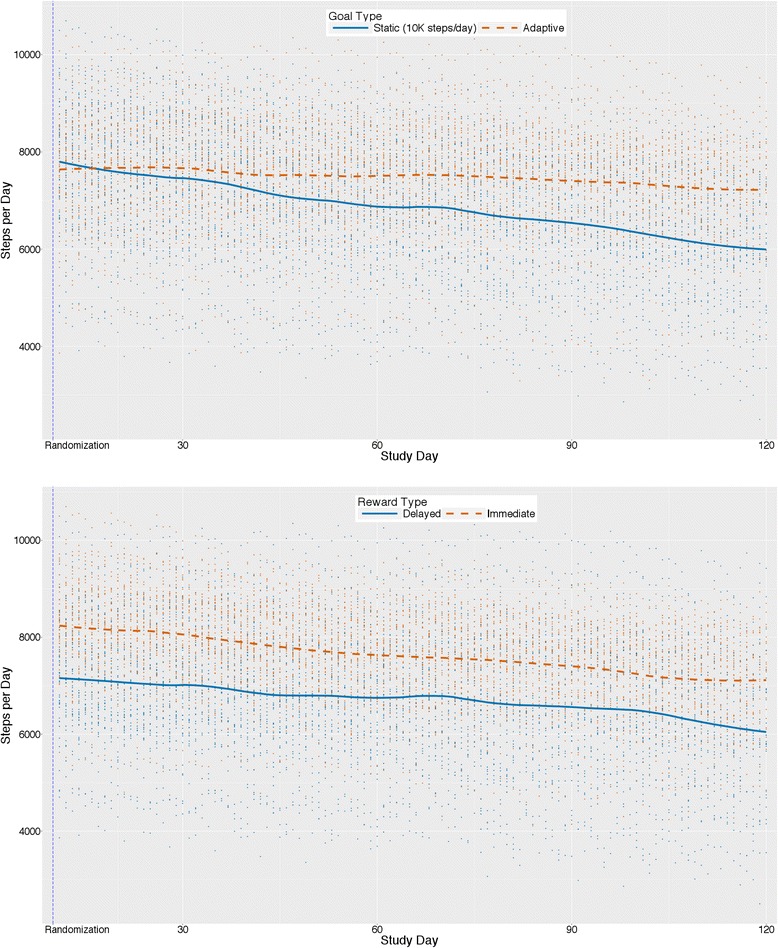
Main effects (*N* = 96) for steps/day by goal type (upper panel) and reward type (lower panel)

Figure 3 shows average trajectories of predicted values after randomization for steps/day separately by type of goal (adaptive vs. static; upper panel) and reward (immediate vs. delayed; lower panel), controlling for the other experimental factor by design and statistically adjusting for covariates. Model-estimated means (not represented in figure) showed that for participants receiving static goals, predicted steps/day increased by 2630 steps/day from baseline (7206 steps/day) to intervention phase (9836 steps/day). This increase was 482 steps/day greater than that for participants receiving adaptive goals (2149-step/day increase; baseline: 7546 steps/day; intervention: 9695 steps/day). The test of this phase x goal interaction, however, was not statistically significant (b = −240.86, 95% CI [−523.54, 41.81], *p* = .095). The rate of change from the beginning to the end of the intervention phase (i.e., from 1 day post-randomization to 110 days post-randomization) did differ significantly between groups (b = 5.33, 95% CI [2.75, 7.91], *p* < .001) with steps/day for participants with adaptive goals decreasing during this period at a slower rate (model-estimated decrease =7.7 steps each day) than for participants receiving static goals (model-estimated decrease =18.3 steps each day).

Participants receiving immediate rewards increased by a model-estimated 2762 steps/day from baseline (7800 steps/day) to the intervention phase (10,562 steps/day), an increase that was 746 steps/day greater than that shown by the delayed rewards group (2016-step/day increase; baseline: 6953; intervention: 8969 steps/day). This difference was significant, (b = 373.12, 95% CI [92.64, 653.60], *p* = .009). The rate of decrease from the beginning to the end of the intervention phase did not differ between reward groups (b = −1.05, 95% CI [−3.61, 1.50], *p* = .418).

At 110 days post-randomization, the predicted mean steps/day value for the adaptive goals group was 1030 steps per day higher than that of the static goals group (b = 515.01, 95% CI [63.50, 966.51], *p* = .028). The predicted mean steps/day value for the immediate rewards group, at 110 days post-randomization, was 1361 steps/day higher than that of the delayed rewards group (b = 680.50, 95% CI [229.70, 1131.30], *p* = .004).

### Group interactions and steps per day

**Fig. 4:**
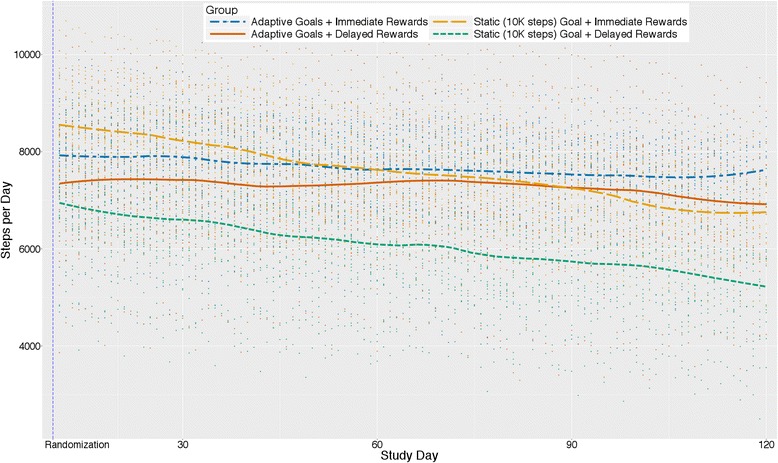
Average change in steps/day by group type and reward type interactions (*N* = 96)

Figure 4 presents trajectories of predicted values from interactions between goal x reward x phase for steps per day. The goal x reward x phase interaction was significant (b = −329.52, 95% CI [−581.84, −77.19], *p* = .010), such that the model-estimated increase (not represented in figure) from baseline to intervention was greater for participants prescribed static goals with immediate rewards (3333-step/day increase from baseline to intervention phase) compared to those for those prescribed adaptive goals with delayed rewards and adaptive goals with immediate rewards (increases of 2105 and 2192 steps/day, respectively), which in turn were stronger increases than that for the static goals with delayed rewards group (increase of 1928 steps/day). There was insufficient power to test goal x reward x intervention day interactions on rate of change post-intervention by subgroup.

### Effects of individual components on MVPA minutes per day

Participants on average increased MVPA by a model-estimated 12.7 min/day from baseline to intervention phase (*p* < .001), and from the beginning to the end of the intervention phase (1 day post-randomization to 110 days post-randomization) there was an average decline (across all groups) of 3.5 *s* each day (b = −0.06, 95% CI [−0.09, −0.02]] for rate of change for MVPA min/day, *p* = .001).

**Fig. 5:**
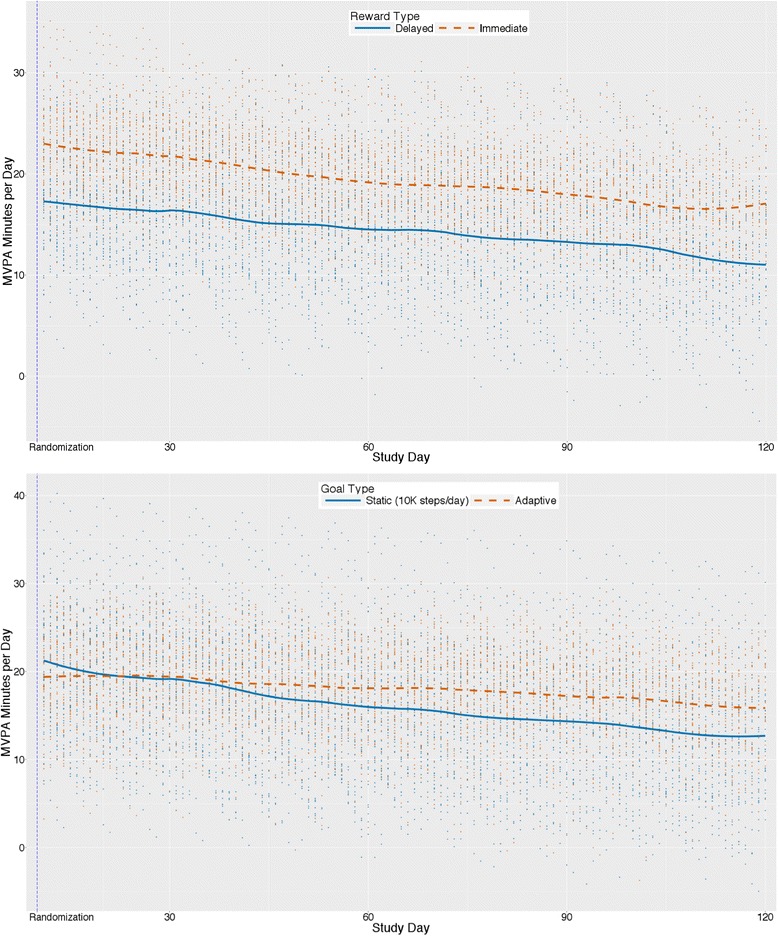
Main effects (*N* = 96) for MVPA minutes/day by goal type (upper panel) and reward type (lower panel)

Figure 5 shows average trajectories for predicted values over 4 months for MVPA minutes/day separately by goal type (adaptive vs. static; upper panel) and reward type (immediate vs. delayed; lower panel) while controlling for the other experimental factor by design and statistically adjusting for covariates. Model-estimated means (not represented in the figure) showed that for participants receiving static goals, predicted MVPA minutes/day increased by 14.0 min/day from baseline (30.1 min/day) intervention phase (44.0 min/day). This increase was 2.5 min/day greater than that for participants receiving adaptive goals (baseline: 31.7 min/day; intervention: 43.2 min/day; increase 11.4 min/day). The test of this phase x goal interaction, however, was not statistically significant (b = −1.27, 95% CI [−2.87, 0.34], *p* = .123). The rate of change from the beginning to the end of the intervention phase (i.e., from 1 day post-randomization to 110 days post-randomization) did differ significantly between groups (b = 0.02, 95% CI [0.01, 0.04], *p* = .004) with MVPA min/day for participants receiving adaptive goals decreasing slower during this period at about half the rate (model-estimated decrease =2.2 s each day) as participants receiving static goals (model-estimated decrease =4.8 s each day).

Participants receiving immediate rewards increased by a model-estimated 15.0 min/day from baseline (32.3 min/day) to the intervention phase (47.3 min/day), an increase that was 4.6 min/day greater than the delayed rewards group’s 10.4-min/day increase (baseline: 29.5 min/day; intervention: 40.0 min/day). This difference was significant (b = 2.29, 95% CI [0.70, 3.88], *p* = .005). The rate of decrease from the beginning to the end of the intervention phase did not differ between reward groups (b = 0.00, 95% CI [−0.02, 0.01], *p* = .659).

At 110 days post-randomization, the predicted mean MVPA min/day value for the adaptive goals group was 3.9 min per day higher than that of the static goals group (b = 1.95, 95% CI [−0.25, 4.15], *p* = .086). The predicted MVPA mean min/day value for the immediate rewards group at 110 days post-randomization was 6.6 min/day higher than that of the delayed rewards group (b = 3.29, 95% CI [1.10, 5.48], *p* = .004).

### Group interactions and MVPA minutes per day

**Fig. 6:**
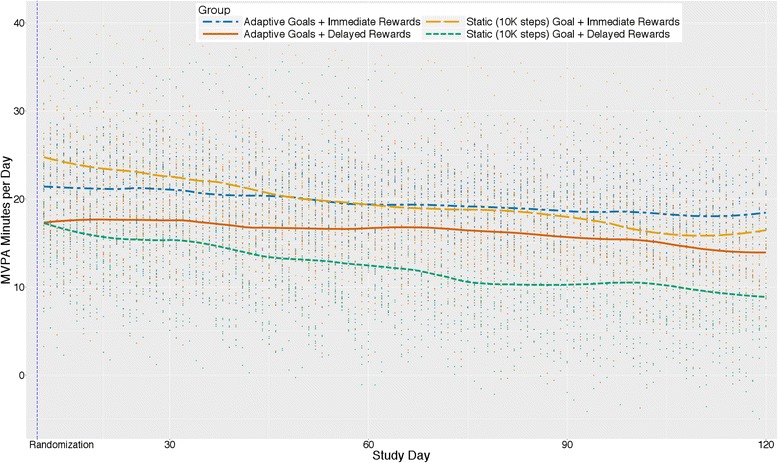
Average change in MVPA minutes/day by group type and reward type interactions (*N* = 96)

Figure 6 presents trajectories of predicted values from the goal x reward x phase interaction for MVPA minutes/day. The goal x reward x phase interaction was significant (b = −1.59, 95% CI [−3.02, −0.15]*p* = .030), such that the static goal with immediate reward group showed a stronger increase from baseline to intervention phase (17.8-min/day increase from baseline to intervention phase) than the adaptive with immediate rewards group (12.1-min/day increase), which, in turn, showed a stronger increase than the adaptive with delayed rewards and static with delayed rewards groups (increases of 10.7 and 10.1 min/day, respectively).

### Incentive amounts

Table 2 presents incentives amounts earned by group status (*N* = 96). None of the participants selected the charity option. The immediate rewards group and the adaptive goals group earned approximately $6.50 more per participant than either the delayed rewards or static goals group, but median and mean amounts across groups were not significantly different (*p* < .05).

**Table 2:** Incentives earned by group status (*N* = 96)

Group status	N	Mean	SD	Median	Sum
Static Goals	47	$62.87	$29.90	$70.00	$2955.00
Adaptive Goals	49	$69.29	$20.18	$70.00	$3395.00
Delayed Rewards	47	$62.77	$17.53	$70.00	$2950.00
Immediate Rewards	49	$69.39	$31.12	$70.00	$3400.00
Total	96	$66.15	$25.48	$70.00	$6350.00

## Discussion

One question that emerged from our previous trials was whether changes to physical activity resulted from goals or reinforcement components alone or an interaction between these two factors [16, 40]. The current factorial trial explored independent and joint contributions of these components for increasing steps/day and MVPA min/day over time.

Most PA interventions (research or commercial) aimed at the public often offer activity monitors and prescribe fixed criterion targets [9] or goals that increase linearly over the course of an intervention [10–12]. These fixed or static goal setting interventions typically do not consider intra-individual variability in PA, especially during the intervention, resulting from real world barriers (e.g., work and school schedules, injury, natural changes in motivation, stress, or ability) or changes in settings (e.g. vacation to lower or higher walkable cities) that may result in either temporary lapses or strengthening of engagement and performance. Adaptive goals, however, can adjust to patterns in behavior resulting from such circumstances or changes in environmental settings. In the current study participants who were prescribed a static goal of 10,000 steps increased their steps/day on average after randomization, but decreased their activity level steeply over the course of the intervention phase. Participants who received adaptive goals did not increase as much after randomization, but rather initially these participants increased more slowly (as seen by a smaller average difference across phases), followed by a much slower rate of decrease during the intervention phase. The adaptive goal group was expected to have a lower increase in steps initially, and expected to have flatter or more positive trajectory compared to those adults provided a goal of 10,000 steps, because adaptive goals as operationalized in this study were always equivalent to the 60th percentile of a participant’s rank-ordered step count over a moving 9-day window. Importantly, the static goal group trajectory crossed and dropped below the activity of the adaptive groups (see Figs. 3a and 5a) within four months, even with immediate rewards (see Figs. 4 and 6). The implication is that adaptive goals will not result in immediate changes as large as static goals, but can contribute to a more gradual and perhaps more sustainable behavior change process over 4 months. It is also possible that adaptive goals result in greater engagement with the behavior change process due to a gamification-like experience or, as hypothesized, a closer match to participant’s individual performance and intra-individual variability, or both. The evidence and theoretical rationale suggest that prescribing adaptive goals may be preferred over static goals (i.e., 10,000 steps/day) for increasing steps/day when possible.

Recently, a combined review and meta-analysis contrasting interventions using various goal setting approaches for physical activity to controls without the use of goal setting found a medium positive effect (Cohen’s d = 0.55) for any type of goal setting, with variability in effect sizes observed across interventions [54]. The meta-analysis identified moderators of the effect size and significant positive effects were evident regardless of the individual who prescribed goals (e.g., practitioners or self), and performance was better for interventions promoting daily over weekly physical activity. The review also noted studies that set weekly and bi-weekly goals seemed to perform better than setting daily goals. However, only a single study with daily goals was included in the meta-analysis. Studies comparing different types of goals to each other (e.g., adaptive vs. static), as seen in the current study, were not included. The current study offers supporting evidence for a novel adaptive goal setting approach. Further research confirming the efficacy of adaptive goal setting is needed for physical activity in larger and longer studies (e.g. 1 year) to verify current findings and to explore longer-term effects of adaptive goal setting. Additionally, adaptive goal setting should be tested with other health behaviors such as reducing sedentary time or increasing frequency of healthful eating behaviors.

Immediate rewards for accomplishing goals resulted in increased physical activity and this finding was independent of the type of goal prescribed. Participants receiving immediate rewards differed by about 746 steps/day on average, and this result was greater than the average change for goal type. The incentive amounts provided for participation only escalated each month (interval schedule) and approximated the estimated amount that could be earned by the immediate reward group (i.e., $45 over 110 days). These results add to the growing body of literature showing that financial incentives successfully promote health behaviors [34, 55–59] and remarkably suggest that immediate rewards as small as one U.S. dollar per day can be leveraged to increased PA among adults with household incomes meeting or exceeding the median household income for the U.S. [60]. Results also suggest that escalating interval rewards for participation only, which are delayed and non-contingent relative to offering daily rewards for goal attainment, are less effective for promoting physical activity, even though many physical activity research studies use such an approach to providing incentives [34, 61, 62]. Use of incentives in such studies can be further leveraged by offering rewards sooner and contingent on goal attainment, thereby maximizing their utility. Our results are also consistent with the literature advocating for “smaller, sooner” as an optimized approach to offering financial incentives for behavior change [20].

Consistent with behavioral economics [20], we rewarded actual behavior change and not intentions to change or changes to weight, and we used “smaller-sooner” rather than “larger-later” incentives. The use of financial rewards to change behavior has been admonished as undermining or crowding-out intrinsic motivation [57]. However, a recent systematic review strongly counters this criticism from both the psychological and economic literatures for health-related behaviors [57]. The review found no evidence to support an a priori position that incentives undermine intrinsic motivation for adoption or maintenance because health behaviors are a class of behaviors without existing high levels of intrinsic motivation. Therefore, the use of incentives to promote health behavior adoption should not be dismissed. Rather, how to optimize delivery of financial rewards for adoption and maintenance of health behaviors, while minimizing any hypothetical or potential side-effects are open empirical questions [20, 55, 56, 58, 59, 63, 64]. Additionally, the estimated cost of using small incentives to promote physical activity (~$45 over 4 months) and a $6.50 difference between groups during the intervention phase in this study should be contrasted with the costs of inactivity. Drugs and financial burdens for chronic diseases related to inactivity that are in excess of $1400/year for obesity and $7383 for diabetes per person per year [65, 66]. Corporate wellness programs [67], government programs [68, 69], and prevention programs [70], have previously paid individuals small amounts for primary prevention and chronic disease–related behavior change. Thus, incentives are already being used for health promotion and optimizing their utility is an important consideration for policy and practice. While the current study provides insight into how incentives could be used more potently to increase PA, further studies are needed to compare against other incentive strategies and to see whether these behavioral outcomes result in disease-related changes.

This study also examined the joint contributions of goals and immediate rewards. Theoreticians have posited that combining measurement, goals, feedback, and rewards for goal attainment thereby creating a perpetual “feedback loop”, as opposed to merely setting a goal alone, can promote behavior change via sophisticated shaping approaches [71–73]. Figure 4 indicated that participants prescribed static goals with immediate rewards for goal attainment had the greatest change on average from baseline followed by participants receiving adaptive goals with immediate rewards, adaptive goals with delayed rewards, and finally static goals with delayed rewards. However, the plots and our analyses also suggest that two groups (i.e., adaptive goals with immediate rewards and adaptive goals with delayed rewards) had a less precipitous decline during the treatment phase. Previous work by Adams et al. observed an initial positive level change for adaptive goals with immediate rewards, with a U-shaped trajectory over a 6-month period [16, 40]. Visualizations of the data here suggest that with delayed rewards, static goals (i.e., 10,000 steps) result in changes to physical activity that returned to levels observed at baseline by the end of 4 months -- implying that small immediate rewards in conjunction with static goals, and adaptive goals with immediate or delayed rewards, should be preferred over static goals with delayed rewards.

### Correspondence between steps/day and MVPA min/day

The main effects and interactions for goal and reward type on MVPA minutes/day were similar to steps/day. A larger change in overall level was observed for immediate compared to delayed rewards and for static compared to adaptive goals. However, as reflected in the pattern observed for steps/day, the adaptive goal group decreased at a slower rate over time suggesting that adaptive goals mitigated decreases in motivation compared to static goals. There was no difference between immediate and delayed rewards groups for change over time from immediate post-randomization to the end of the intervention phase. These results suggest that participants likely increased their intensity of movement, even though the goals and rewards targeted steps (i.e., a metric without an inherent intensity). However, MVPA was estimated from the Fitbit using a cadence of ≥100 steps per minute; an independent accelerometer was not used to verify this result.

### Methodological considerations

Strengths of the study include a factorial, randomized design with groups matched on several intervention components such as a Fitbit for the duration of the study, one-time educational materials, prompt-to-action texts, incentive amounts, and communication mode. These components can be eliminated as alternative explanations for the differences observed between groups. The study untangled unique and joint effects for goal setting and reward factors for promoting activity with activity monitors. Studies comparing packaged multi-component treatments against a measurement-only control group do not allow for identification of active components driving behavior change or development of optimized treatment packages. An additional strength was the use of an intent-to-treat analysis without imputation. These strengths support confidence in the internal validity for the observations. Limitations should be noted and include a convenience sample of English speaking, insufficiently active, yet otherwise healthy, overweight and obese men and women with daily access to the Internet. However, similar to many behavioral interventions, less than a quarter of the sample was men limiting generalizability to this subgroup. Another limitation was that physical activity volume (mainly walking) was promoted over a specific intensity of activity and wear time could not be examined as a covariate. The interventions targeted steps because the Fitbit Zip displayed steps to participants, which allowed participants to monitor their daily progress. Regardless, it appears from the cadence analyses that participants did increase their MVPA minutes during the study. One might speculate that participants figured out our adaptive goal setting algorithm and attempted to “game the system.” At follow-up, we asked participants in the adaptive goal group, “Did you ever try to figure out how we calculate new goals? If yes, can you tell us how we do it?” While several participants offered affirmative answers, none could describe our adaptive goal setting algorithm correctly. No such “gaming” patterns were observed in any individual’s data across any of the groups either, mitigating this concern. Lastly, the intervention period was limited to approximately 4 months. A longer duration intervention may allow for adaptive goals to promote further improvements. Our previous study found a U-shaped trajectory over 6 months with a turning point around months three and four. Future directions could explore adaptive goal setting with immediate positive reinforcement interventions focusing on increasing MVPA minutes per day, in larger and longer studies, with more diverse samples including clinical and healthy populations to improve fitness levels.

## Conclusions

Adaptive goals outperformed static goals (i.e., 10,000 steps) and small immediate rewards outperformed larger, delayed rewards over 4 months. Use of delayed rewards with static goals resulted in changes to physical activity that approximated baseline levels by the end of the study. Results suggest that use of static goals require small immediate rewards to be effective over time. Adaptive goals with either immediate or delayed rewards should be a preferred approach over static goals with delayed rewards.

## Abbreviations

ICC: Intra Class Correlations
m/s: Meters per second
min/day: Minutes per day
MVPA: Moderate-to-vigorous physical activity
PA: Physical Activity
Steps/min: Steps per min
