# Erratum to: Adaptive goal setting and financial incentives: a 2 × 2 factorial randomized controlled trial to increase adults’ physical activity

**DOI:** 10.1186/s12889-017-4231-x

**Published:** 2017-04-06

**Authors:** Marc A. Adams, Jane C. Hurley, Michael Todd, Nishat Bhuiyan, Catherine L. Jarrett, Wesley J. Tucker, Kevin E. Hollingshead, Siddhartha S. Angadi

**Affiliations:** 1 College of Health Solutions, Arizona State University, 425 North 5th Street (MC9020), Phoenix, AZ 85004 USA; 2 College of Nursing and Health Innovation, Arizona State University, 500N. 3rd Street, Phoenix, AZ 85004 USA; 3 Global Institute of Sustainability (GIOS), Arizona State University, Tempe, AZ 85287 USA

## Erratum

Following publication of this article [1], it has come to our attention that in Fig. 5 the top and bottom panels were not presented in the correct order. Reward Type was presented in the top panel and Goal Type in the bottom panel, which is incorrect and inconsistent with the figure title and presentation in text.
